# Characterisation of the First Complete Dengue Genome in Sierra Leone

**DOI:** 10.3390/v18030298

**Published:** 2026-02-28

**Authors:** Allan K. O. Campbell, Ifeanyi Omah, Andy M. Diouf, Mignane Ndiaye, Julian S. O. Campbell, Edyth Parker, Vidalyn Folorunso, Anu J. Williams, Mattu T. Kroma, Sia Y. Mani, Naomi Daniel-Sesay, Zein Souma, Choe Miller, Roberta Lansana, Amanda M. Kargbo, Fay Chalobah, Mamadou Cisse, Mamadou Malado Jallow, Joseph Charles, Aminata T. Koroma, Joseph Sam Kanu, Abebaw Kebede, Collins Tanui, Sofonias Tessema, Oumar Faye, Gamou Fall, Ndongo Dia, James S. Squire, Mohamed Boie Jalloh, Mohamed Alex Vandi, Zikan Koroma, Abdourahmane Sow, Foday Sahr, Bocar Sow, Doris Harding, Boubacar Diallo

**Affiliations:** 1 Central Public Health Reference Laboratory, National Public Health Agency, Freetown P.O. Box 529, Sierra Leone; juliansocampbell@yahoo.com (J.S.O.C.); vidish27@gmail.com (V.F.); anuwilliams500@gmail.com (A.J.W.); mattutetoh23@gmail.com (M.T.K.); siayankosamani9@gmail.com (S.Y.M.); contehhusinatu@gmail.com (N.D.-S.); zeinsouma1993@gmail.com (Z.S.); choe063@gmail.com (C.M.); robertalansana3@gmail.com (R.L.); amandamalinda8@gmail.com (A.M.K.); 2 National Public Health Agency, Ministry of Health, Freetown P.O. Box 529, Sierra Leone; rfaycecilia@yahoo.co.uk (F.C.); josephcharles785@yahoo.co.uk (J.C.); aminata_krm@yahoo.com (A.T.K.); samjokanu@yahoo.com (J.S.K.); jmssquire@yahoo.com (J.S.S.); mboie1537@gmail.com (M.B.J.); mohamedavandi69@gmail.com (M.A.V.); zikankoroma@gmail.com (Z.K.); fsahr65@gmail.com (F.S.); dorisharding@yahoo.com (D.H.); 3 Faculty of Medical Laboratory Sciences and Diagnostics, College of Medicine and Allied Health Sciences, Freetown P.O. Box 529, Sierra Leone; 4 Institute of Ecology and Evolution, University of Edinburgh, Edinburgh EH9 3FL, UK; ifeanyi.omah@ed.ac.uk; 5 Institut Pasteur de Dakar, Dakar 22000, Senegal; andydiouf@gmail.com (A.M.D.); mignane.ndiaye@pasteur.sn (M.N.); mamadou.cisse@pasteur.sn (M.C.); mamadoumalado.jallow@pasteur.sn (M.M.J.); oumar.faye@pasteur.sn (O.F.); gamou.fall@pasteur.sn (G.F.); ndongo.dia@pasteur.sn (N.D.); abdourahmane.sow@pasteur.sn (A.S.); bocar.sow@pasteur.sn (B.S.); boubacar.diallo@pasteur.sn (B.D.); 6 Institute of Genomics and Global Health, Redeemer’s University, Ede 232101, Nigeria; 7 Africa Centres for Disease Control and Prevention, Addis Ababa 1000, Addis Ababa, Ethiopia; kebedeab@africacdc.org (A.K.); tanuic@africacdc.org (C.T.); sofoniast@africacdc.org (S.T.)

**Keywords:** dengue virus, DENV-2, Sierra Leone, arbovirus surveillance, phylogenetics, genomic epidemiology

## Abstract

Dengue is the leading mosquito-borne viral cause of human illness and death. More than four billion people globally are at risk of dengue virus (DENV) infection, and most infections are asymptomatic or present with a non-specific febrile illness. We characterise the first complete DENV-2 genome from Sierra Leone, recovered from a febrile adult who tested RT-PCR–positive. The sequence was identified as DENV-2 genotype II, lineage F.1.1. Phylogenetically, the Sierra Leone genome formed a well-supported sister lineage with a 2024 USA DENV-2 genome; both were nested within but clearly diverged from Indian DENV-2 sequences (2021–2022) and were distinct from the Réunion DENV-2 clade. The degree of genetic divergence was incompatible with a recent or direct import of a South Asian lineage and was more consistent with diversification in an under-sampled Indian Ocean/South Asia network or outside this region in Africa. With a single Sierra Leone genome, the source and extent of local transmission remain unresolved. These findings underscore the benefits of integrating differential diagnostics and genomics into routine care for febrile illness and sustaining regional arboviral surveillance.

## 1. Introduction

Dengue is the leading mosquito-borne viral cause of human illness and death. More than four billion people globally are at risk of dengue virus (DENV) infection, and most infections are asymptomatic or present with a non-specific febrile illness [1]. Each year, ~96 million symptomatic cases are recorded, with ~500,000 progressing to severe disease with ~400,000 deaths [2]. Four distinct serotypes (DENV-1–4) are circulating globally and differ in their epidemic potential and clinical severity [3]. In Africa, confirmed outbreaks and co-circulation of multiple serotypes, including DENV-2, have been reported in Senegal, Mauritania, Cabo Verde, Burkina Faso and elsewhere, with entomological evidence of active transmission in urban settings [4,5]. This suggests that DENV is widespread across the continent, even in contexts where access to differential diagnostics is limited.

Outside malaria, which is often the only routinely available point-of-care test, most non-malarial fevers are empirically managed, leaving their aetiologies largely uncharacterised and creating substantial gaps in policy responses [6]. However, where differential testing has been deployed, substantial under-detection emerges: in a cohort of 515 febrile patients in southern Nigeria who were malaria- and typhoid-negative, ~28% had evidence of prior DENV infection, ~8% showed recent/ongoing infection, and ~3% met criteria for acute infection [7]. This diagnostic gap obscures the burden of arboviruses such as dengue and constrains timely outbreak detection. More broadly, in West and Central Africa (WCA), the epidemiology of dengue remains under-sampled, even as rapid urbanisation, mobility, and climate change are reshaping *Aedes* mosquito ecology and likely accelerating transmission potential [8].

Historical dengue reporting in Sierra Leone is sparse and fragmented, with no sustained nationwide arboviral surveillance and very limited routine testing beyond malaria [9,10]. Given the established presence of *Aedes aegypti*, densely populated urban cities (Western Area Urban), porous regional travel networks, and the increasing ecological suitability resulting from climate variability [9,11], undetected endemic or recurrently imported DENV transmission is plausible. A seroprevalence study conducted at Kenema District Hospital, Sierra Leone, revealed that dengue viruses were the aetiology of fever in 117 patients out of 149 (78.52%), with evidence of exposure to all four serotypes [9]. However, in the absence of systematic, syndromic, and laboratory-supported surveillance, the magnitude, serotype diversity, and transmission dynamics in Sierra Leone remain uncertain.

To address this gap, Sierra Leone implemented the Syndromic Sentinel Surveillance Strategies (4S) in June 2025, adapted from the Integrated Surveillance and Laboratory Network (RISLNET) model in Senegal, where 4S has repeatedly enabled early detection of respiratory and arboviral outbreaks, including Rift Valley fever (RVF), Crimean–Congo hemorrhagic fever (CCHF), Zika, yellow fever, chikungunya, and dengue, since 2015 [5,12]. 4S links frontline clinical syndromes to targeted laboratory testing and genomic confirmation, creating an operational platform for rapid risk assessment. Here, we report the genomic characterisation of the first laboratory-confirmed dengue case detected through this system, and we place this finding within the regional context of evolving DENV transmission. Our results underscore the need and feasibility of integrating differential diagnostics and genomic surveillance into routine care for febrile illness to understand the true burden of dengue and guide public health action in Sierra Leone.

## 2. Materials and Methods

### 2.1. Sentinel Sites

During the implementation phase, two pilot sites were selected for the establishment of the sentinel surveillance: Christ the King Hospital (CKH) in Waterloo and Kenema Government Hospital (KGH) in Kenema (Figure S1). The CKH, located in the Western Rural Area district, has a large population catchment, while in Kenema, previously published studies highlighted the circulation of arboviruses. These studies in Kenema identified DENV and Chikungunya virus as causes of febrile illness among patients [13]. In addition, DENV was diagnosed as the aetiology of dengue fevers among 77% of patients at a clinic in Kenema, and serological evidence of infection by at least one DENV serotype was detected in 78.52% (117/149) of human serum samples collected in Kenema [9]. During the practical phase, staff from the selected sites were trained in syndromic surveillance, including case definition; completing the case identification form; specimen sampling from suspected cases; storage, packaging, and transport; and data entry on a digital health platform.

Patients were considered suspect for arbovirus infection if they presented with fever (>38 °C) and at least 2 minor signs, such as headache, muscle pain, joint pain, retro-orbital pain, or skin rash. For each suspected case, a blood sample was taken. From the start of implementation, all samples collected as part of this surveillance were sent weekly to the Central Public Health Reference Laboratory.

### 2.2. Central Public Health Reference Laboratory

Building on its extensive experience in disease surveillance and control, CPHRL was selected as the national reference laboratory to test suspected cases. RT-PCR reagents were provided for testing human serum samples for seven arboviruses of public health concern in West Africa, including Crimean–Congo haemorrhagic fever, chikungunya, dengue, Rift Valley fever, West Nile, yellow fever, and Zika. Practical sessions were conducted with staff who previously had considerable experience in molecular biology. This included the pre-analytical phase, which covers sample reception, handling, and storage; the analytical phase; and the post-analytical phase, with results reported on the digital health platform.

### 2.3. Sample Collection, Nucleic Acid Extraction, and Next-Generation Sequencing

Blood samples were collected at Christ the King Hospital (CKH) during the case investigation and transported at 2–8 °C to the CPHRL for molecular detection and subsequent sequencing. Serum was separated from the collected blood, and total viral nucleic acid was extracted using the MagMAX Viral/Pathogen Nucleic Acid Isolation Kit on the KingFisher Automated Extraction System (both from Thermo Fisher Scientific, Waltham, MA, USA), following the manufacturer’s protocols.

A RT-PCR assay was carried out using Lightmix Polymerase 1-step RT-PCR mix (TIB MOLBIOL, Berlin, Germany). The reaction mix consisted of 5 µL of viral RNA, Lightmix buffer, and 10 µM of previously published dengue-specific primers that detect all four serotypes [14]. Samples were first confirmed by real-time reverse transcription PCR (RT-PCR), with a cycle threshold (Ct) value < 30.

Library preparation was performed using the Illumina RNA Prep with Enrichment (L) Kit(Illumina, Inc., San Diego, CA, USA), incorporating the Viral Surveillance Panel (VSP) 2.0, which targets epidemic-prone pathogens, including DENV. Following hybridisation, libraries were captured using streptavidin-coated magnetic beads and then amplified, purified, and quantified. Quality assessment was conducted using the Qubit fluorometer (Thermo Fisher Scientific, Waltham, MA, USA) and the Agilent TapeStation system (Agilent Technologies, Santa Clara, CA, USA). Sequencing was performed on the Illumina MiniSeq platform, generating 2 × 150 bp paired-end reads.

### 2.4. Bioinformatics Analysis

#### 2.4.1. Genome Assembly

We used a reference-guided assembly pipeline to analyse the sequencing data generated for the DENV-2 using the Illumina Viral Surveillance Panel v2 kit (Illumina, Inc., San Diego, CA, USA). Raw reads were quality-filtered with Trimmomatic v0.39 [15] (parameters: ILLUMINACLIP:2:30:10 SLIDINGWINDOW:4:20 MINLEN:50) to remove adapters and low-quality bases. To deplete host material, filtered reads were aligned to the human genome (hg38) with Bowtie2 v2.5.4 [16] unmapped reads were retained for viral analysis. These de-hosted reads were then mapped with Minimap2 v2.28 [17] to representative references for the four dengue serotypes: DENV-1 (NC_001477.1), DENV-2 (NC_001474.2), DENV-3 (NC_001475.2), and DENV-4 (NC_002640.1). Assemblies achieved a high breadth of coverage (up to ~97% for DENV-2 in our dataset). Viral species/serotype assignment was corroborated by BLASTn (2.17.0+) against the NCBI nucleotide database [18].

#### 2.4.2. Variant Calling and Consensus Generation

Variants were called with iVar v1.4.3 [19]. We required per-site depth ≥ 50×; positions below this threshold were excluded from variant reporting and masked during consensus generation. Consensus genomes were produced with iVar under the same depth filter. Lineages for the resulting consensus sequences were assigned using Nextclade [20].

### 2.5. Phylogenetic Analysis

We combined our one high-quality genome (breadth of coverage > 90%) with publicly available DENV sequences from GenBank and GISAID (*n* = 5575; deliberately enriched for DENV-2) to contextualise likely geographic origins and circulation history. Sequences were aligned with MAFFT v7.52 [21], and a maximum-likelihood tree was inferred with IQ-TREE v2.2.5 [22] using ModelFinder Plus [23] for model selection and ultrafast bootstrap support [24] (UFBoot; 1000 replicates). For visualisation of the results, we subsampled 336 genomes to retain temporal and geographic breadth while reducing redundancy (giving priority to the nearest phylogenetic neighbours of the Sierra Leone genome and to diverse regional representatives).

We reconstructed the time-resolved phylogeny using BEASTx v10.5 [25,26] and used the GTR substitution model with gamma-distributed rate variation across all sites. We employed the uncorrelated relax clock lognormal using a constant coalescent tree prior. Then, we combined two independent MCMC chains of 100 million states run with the BEAGLE computational library [27] Parameters and trees were sampled every 10,000 steps, with 10% of steps discarded as burn-in. Convergence and mixing of the MCMC chains were assessed with Tracer v.1.7.2 [28] and all estimated parameters had effective sample sizes greater than 200.

## 3. Results

### 3.1. Sierra Leone’s Dengue Isolate Is a Member of Genotype II Major Lineage F

A 64-year-old female herbalist presented on 5 July 2025 with five days of headache and arthralgia. She had no haemorrhagic signs or shock and was suspected of having early dengue. Blood samples were sent to the Central Public Health Reference Laboratory, where RT-PCR confirmed dengue virus infection. However, a thorough epidemiological investigation could not be conducted due to a concurrent mpox outbreak in Sierra Leone at the time [29].

**Figure 1:**
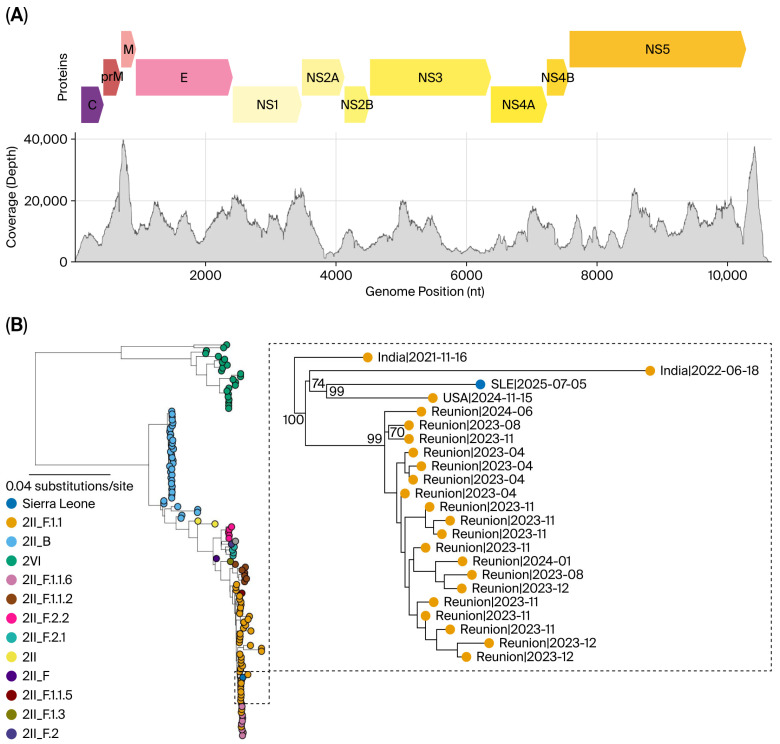
Genome characterisation and the Maximum-likelihood phylogeny placing the Sierra Leone DENV-2 genome in the global context. (**A**) Coverage of the assembled genome. (**B**) Tree of representative DENV-2 viruses coloured by minor lineages; the Sierra Leone genome 064_S23|SLE|2025 is highlighted in blue. The solid box expands the relevant DENV-2 lineage 2II_F.1.1 clade. Within this clade, two Indian genomes from 2021 to 2022 are basal to the Sierra Leone genome, followed by a sister clade comprising a tight cluster from Réunion Island, sampled between 2023 and 2024. Tip labels display accession, location, host, and date. Branch lengths represent substitutions per site (with 100% bootstrap support). The topology places the Sierra Leone virus within DENV-2 2II_F.1.1, nearest to Indian Ocean/South Asia lineages and adjacent to the Réunion outbreak cluster.

To identify the DENV serotype, we generated a near-complete DENV genome from the Sierra Leone case. The consensus sequence had a genome coverage of 99.7% (Figure 1A). BLAST analysis identified the closest matches as DENV serotype 2 (DENV-2) sequences sampled in India between 2021 and 2022, with nucleotide similarities ranging from 99.23% to 99.45%. Using Nextclade [20], the sequence was assigned to genotype II, major lineage F, and minor lineage 1.1 (2II_F.1.1) [30]. Also known as the Cosmopolitan genotype, DENV-2 genotype II is one of the most widely distributed and diverse DENV genotypes [30].

To place our new sequence within the context of global DENV diversity, we inferred a maximum-likelihood phylogeny from 5475 global DENV-2 genomes. Our SLE sequence formed a well-supported cluster (bootstrap support = 99) with a 2024 USA genome. Phylogenetically, this cluster was nested within, yet distinct from, lineages sampled in India. Notably, the SLE sequence differs from the closest USA sequence by 64 substitutions and from Indian relatives (2021–2022) by 127 substitutions, with a substantial branch length (45 substitutions) extending from the common ancestor (Figure 1B). Additionally, the SLE sequence formed a sister lineage to sequences sampled from Réunion Island (2023–2024) (bootstrap support = 99), separated by 66 substitutions. Réunion has experienced recurrent DENV epidemics since 2017, driven by repeated introductions from the Indian Ocean region [31,32]. However, the combination of the tree topology, significant genetic divergence and the patient’s lack of travel history suggested that undetected transmission chains are circulating within Africa. While the lineage ultimately originated from the South Asia/Indian Ocean network, the data supported an extended period of local or regional diversification, incompatible with a recent direct introduction event involving India, the USA, or Réunion.

### 3.2. Sierra Leone’s Dengue Isolate Started to Diverge from the South Asia Relative Since at Least 2021

**Figure 2:**
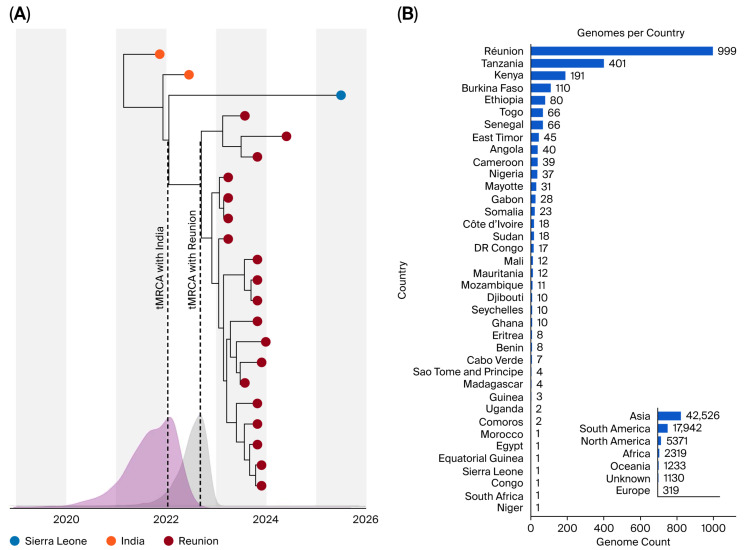
Estimating the divergence time between the SLE sequence and other 2II_F.1.1 diversity. (**A**) Highest Independent Posterior Subtree Reconstruction (HIPSTR) phylogeny of DENV-2 with absolute time on the x-axis. The Indian diversity (orange) is basal to the Sierra Leone genome (blue), which forms a sister lineage to the Réunion Island cluster (dark red). Probability density plots below show the estimated time to the most recent common ancestor (tMRCA) for the SLE strain against Indian (purple) and Réunion (grey) lineages. (**B**) Count of available DENV genomes per African country on GISAID. The inset compares global counts by continent, highlighting the significant surveillance gap in Africa.

Bayesian phylogenetic reconstruction was used to estimate the time of divergence between the SLE sequence and the 2II_F.1.1 diversity found in India and Réunion. We estimated the time to the most recent common ancestor (tMRCA) to be December 2021 [95% HPD: November 2020–October 2022] relative to the closest South Asian strains (Figure 2A). This four-year temporal gap argues against a recent import of a currently circulating South Asian variant.

Identifying the exact route of entry is challenged by the limited availability of DENV genomes across Africa (Figure 2B). It remains unclear whether the virus diverged locally within South Asia before a historical introduction, or if the divergence occurred during cryptic circulation within Africa. However, given the patient’s lack of travel history, the data strongly support unsampled transmission, potentially within Sierra Leone or a neighbouring country, originating from an introduction well before the current case was detected.

With only a single SLE genome, the scale of this local transmission remains unknown. Further sampling in West Africa and other parts of Africa is critical to understanding DENV diversity and differentiating between sporadic introductions and established, unsampled lineages.

## 4. Discussion

This study provides the first genomic characterisation of DENV-2 in Sierra Leone, confirming the local presence of lineage 2II_F.1.1. Detected shortly after the launch of the 4S strategy, this finding underscores the necessity of differential diagnostics for non-malarial febrile illness [9,29,33]. The phylogenetic placement of this genome, nested within but distinct from South Asian and Indian Ocean diversity, suggests an extended period of unsampled transmission within the region rather than a recent importation. This distinction is vital; it implies that neighbouring countries to Sierra Leone may harbour an established, unsampled reservoir of DENV-2, given this patient’s recent travel history. Given the absence of multiple contemporaneous genomes from Sierra Leone and the lack of associated phenotypic or epidemiological data, we considered it inappropriate to speculate on selection pressures or transmission pathways based on this single genome.

The geographic expansion of dengue in West Africa poses a significant public health threat. Recent outbreaks in Burkina Faso, Senegal, and Cabo Verde have demonstrated the severity of multi-serotype circulation [12,34] The identification of DENV-2 in Sierra Leone indicates that the country is not isolated from these regional transmission dynamics. Consequently, moving beyond reactive case detection to sustained genomic surveillance is critical. Integrating molecular screening into routine febrile illness management will enable early detection of viral evolution and the potential introduction of new serotypes, thereby informing timely public health interventions [35,36].

Together, these findings extend beyond cataloguing a single genome. They illustrate three converging dynamics: (i) dengue’s increasing reach into West Africa through global pathways, (ii) the critical importance of diagnostic strengthening to avoid underestimation of its true burden, and (iii) the role of genomic surveillance in identifying both introductions and potential molecular adaptations. A comprehensive public health strategy for Sierra Leone should therefore integrate classical surveillance with targeted genomic characterisation, healthcare provider education, and regional collaboration to enable comparative analysis of dengue virus genomes across West Africa and improve interpretation of circulating serotypes and lineages. This integrated approach will be essential for detecting introductions early, mitigating outbreaks, and contributing to a more accurate understanding of dengue’s evolving role in Africa’s infectious disease landscape.

## Data Availability

The dengue virus serotype 2 (F.1.1) genome sequence from this case report has been deposited in the GISAID database (Accession ID: EPI_ISL_20179260).

## Supplementary Materials

The following supporting information can be downloaded at https://www.mdpi.com/article/10.3390/v18030298/s1. Figure S1: Map depicting the 2 sentinel sites in Waterloo, Western Area, and Kenema.
