# Supplementary material for: Characterisation of the First Complete Dengue Genome in Sierra Leone
Source: Viruses. 2026 Feb 28;18(3):298. doi: 10.3390/v18030298 (PMC13029902; doi:10.3390/v18030298)
Supplement: Supplementary file 1 [file viruses-18-00298-s001.zip › viruses-4063922-Supplementary.pdf]

Supplementary figures

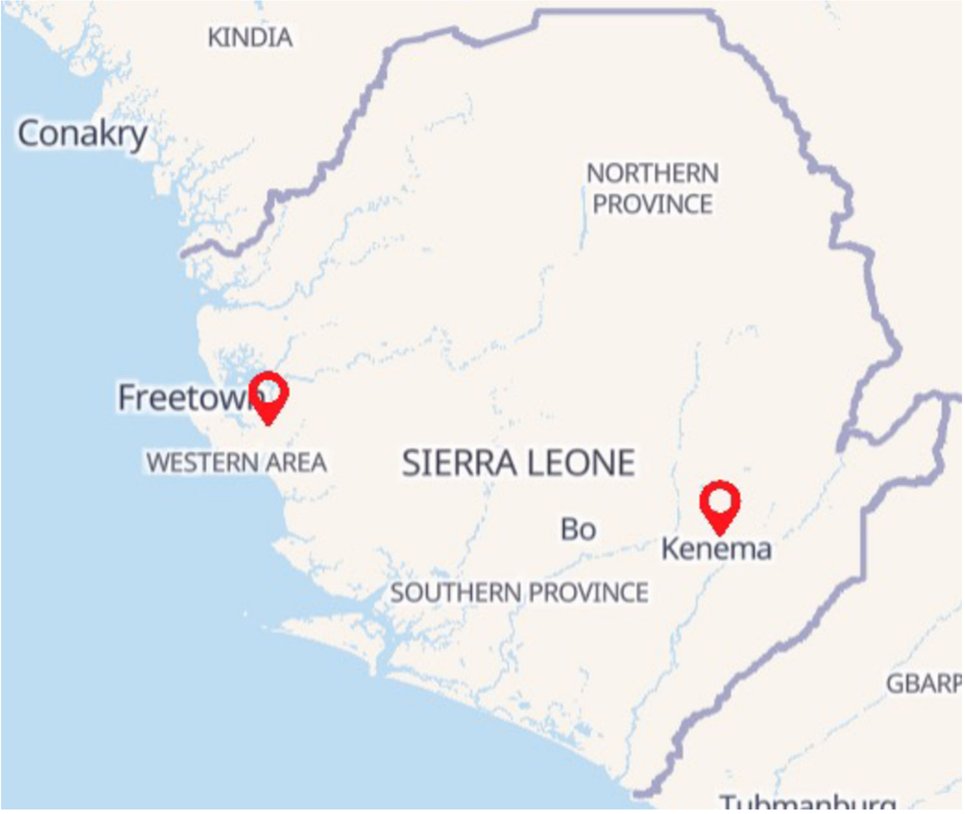

Figure S1: Map describing the 2 sentinel sites in Waterloo, Western Area and Kenema
